# Human Endogenous Retroviruses in Neurodegenerative Diseases

**DOI:** 10.3390/genes15060745

**Published:** 2024-06-05

**Authors:** Gabrielle L. Adler, Kelvin Le, YuHong Fu, Woojin Scott Kim

**Affiliations:** 1 Brain and Mind Centre, The University of Sydney, Sydney, NSW 2050, Australia; 2 School of Medical Sciences, The University of Sydney, Sydney, NSW 2050, Australia; 3 School of Biomedical Sciences, University of New South Wales, Sydney, NSW 2052, Australia

**Keywords:** human endogenous retrovirus, retrotransposon, neurodegenerative diseases, multiple sclerosis, amyotrophic lateral sclerosis, motor neuron disease, frontotemporal dementia, Alzheimer’s disease, Parkinson’s disease, clinical trials, biomarkers

## Abstract

Human endogenous retroviruses (HERVs) are DNA transposable elements that have integrated into the human genome via an ancestral germline infection. The potential importance of HERVs is underscored by the fact that they comprise approximately 8% of the human genome. HERVs have been implicated in the pathogenesis of neurodegenerative diseases, a group of CNS diseases characterized by a progressive loss of structure and function of neurons, resulting in cell death and multiple physiological dysfunctions. Much evidence indicates that HERVs are initiators or drivers of neurodegenerative processes in multiple sclerosis and amyotrophic lateral sclerosis, and clinical trials have been designed to target HERVs. In recent years, the role of HERVs has been explored in other major neurodegenerative diseases, including frontotemporal dementia, Alzheimer’s disease and Parkinson’s disease, with some interesting discoveries. This review summarizes and evaluates the past and current research on HERVs in neurodegenerative diseases. It discusses the potential role of HERVs in disease manifestation and neurodegeneration. It critically reviews antiretroviral strategies used in the therapeutic intervention of neurodegenerative diseases.

## 1. Introduction

DNA units that mobilize from one genomic location to another are known as transposable elements (TEs) [1]. TEs comprise almost half of the human genome and are divided into two classes based on the mechanism of translocation. The majority of TEs are retrotransposons, which mobilize via a ‘copy-paste’ mechanism involving reverse transcription and an RNA intermediate. The minority of TEs are DNA transposons, which mobilize via a simple ‘cut-paste’ mechanism [1,2,3]. Retrotransposons are further categorized into three subtypes: long interspersed nuclear elements (LINEs), short interspersed nuclear elements (SINEs), and long terminal repeats (LTRs).

The physiological importance of TEs is poorly understood, and initially, TEs were considered non-functional ‘junk’ DNA with little physiological relevance. In fact, all DNA transposons and most retrotransposons in the genome were thought to be non-mobile and inactive [1,4]. However, recent studies have posited the role of retrotransposons in genetic diversity and evolution, and as regulatory sequences that tightly control gene expression under certain physiological conditions [1,5,6]. Environmental perturbations are thought to also trigger aberrant expression of retrotransposons, causing DNA damage or increases in episomal DNA. These have led to the idea that dysregulation of retrotransposons is pathologically linked to human diseases, including cancer and autoimmune diseases [1,7,8].

**Figure 1:**
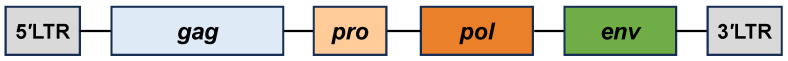
Schematic structure of human endogenous retrovirus genome. gag encodes capsid, nucleocapsid, and matrix protein; pro encodes protease; pol encodes reverse transcriptase; and env encodes envelope protein. LTR, long terminal repeat.

In recent years, increasing knowledge about retrotransposons has driven interest in their potential as a causative factor in the development and progression of neurodegenerative diseases and ageing. Of specific interest are human endogenous retroviruses (HERVs), which belong to the LTR subtype. Phylogenetically, HERVs are members of the *Retroviridae* family, which are retroviruses that have integrated into the human genome via an ancestral germline infection. HERVs are classified into three main groups based on sequence information: gammaretroviruses, betaretroviruses, and spumaretroviruses. The potential importance of HERVs is underscored by the fact that they comprise approximately 8% of the human genome [9]. Structurally, HERVs are constituted of gag, pol, and env genes and two LTRs (Figure 1). The gag gene encodes capsid, pro encodes protease, pol encodes reverse transcriptase, and env encodes envelope protein. The two LTRs act as promoters or enhancers that regulate transcription. However, many, if not most, HERVs do not encode functional proteins, because of various genomic rearrangements, such as deletions and truncations. Interestingly, a recent study provided evidence that dormant HERVs in the human genome could be “awakened” and contribute to the aging process [10]. It was shown that in human senescent cells, the viral genes were transcribed to produce retrovirus-like particles. These particles were shown to induce senescence in young cells, providing a potential target to alleviate ageing [10].

Recent studies have also described the purported role of various HERVs in neurodegenerative diseases, including multiple sclerosis, amyotrophic lateral sclerosis, frontotemporal dementia, Alzheimer’s disease, and Parkinson’s disease. Hence, this review provides an up-to-date review of the relationship between HERVs and neurodegenerative diseases. In doing so, we highlight the potential role of HERVs in neurodegenerative processes, and canvass the idea that HERVs are potential targets for diagnosis of, and therapy for, neurodegenerative diseases.

## 2. HERVs in Multiple Sclerosis

Multiple sclerosis (MS) is a neurodegenerative disease involving autoimmune demyelination of the central nervous system (CNS) [11]. Clinical presentations of MS vary depending on central lesion sites; however, common symptoms include and range from vision problems, sensory impairment, urinary incontinence, motor dysfunction, gait disturbances, and incoordination [12,13]. The disease course of MS varies considerably and is categorized as either relapsing-remitting, secondary progressive, primary progressive, or progressive relapsing MS [12]. MS presents with significant health challenges and is the most common cause of non-traumatic neurological disability affecting young adults, commonly diagnosed in patients aged between 20 and 49 years [11,14]. Epidemiologically, MS is estimated to affect 2.8 million people worldwide (35.9 per 100,000 population), and has female-dominant distribution [15,16]. However, due to the heterogeneity of MS presentation with disparate disease courses, MS diagnosis and treatment remains a difficult challenge [17].

MS pathophysiology involves multiple foci of chronic inflammation from autoimmune demyelinating events, which results in neurodegeneration through axonal damage and reactive gliosis [18]. Neuropathologically, areas of demyelination and neurodegeneration reveal as plaques, common in regions including the optic nerves, spinal cord, brainstem, cerebellum, and white matter regions [18,19]. The etiology of MS is currently unknown; however, some environmental factors have been linked with disease susceptibility. These include vitamin D or ultraviolet B exposure, obesity, smoking, and infection by the Epstein–Barr virus [15]. It has also been proposed that MS could be caused by HERVs [20].

The first report of the involvement of a human retrovirus in MS patients was by Perron and colleagues [21], in which retroviral-like particles with reverse transcriptase activity were detected in MS cerebrospinal fluid (CSF). The particles were characterized as an MS-associated retrovirus, a member of the HERV-W family [22]. Moreover, multiple studies have shown that HERV-W mRNA expression in MS brain lesions and plaques, peripheral blood mononuclear cells (PBMCs), and CSF is elevated compared to healthy controls [23,24,25,26,27,28,29,30,31,32,33]. Furthermore, levels of HERV-W transcripts are associated with the severity of MS [34]. HERV-W env protein is frequently found in MS brain lesions [23,28,35,36]. Moreover, HERV-W gag protein is present in endothelial cells and env protein in macrophages in MS lesions [36], and HERV-W pol is expressed in the peripheral blood, CSF, and brain lesions of MS patients [37]. Other HERVs are also associated with MS, including HERV-K18 [38,39], HERV-Fc1 [40], HERV-H, HERV-K, HERV-E, and HERV-15 [37,41].

The potential pathogenicity of HERV-W was shown in an experimental autoimmune encephalomyelitis mouse model, in which dose-dependent clinical manifestations were observed upon exposure to HERV-W env protein [42]. A more recent study showed that patients with a higher expanded disability status scale (EDSS) score and relapsing conditions had increased HERV-W env active loci in PBMCs compared to patients with low EDSS scores and healthy controls [43], suggesting that HERV-W load is associated with disease severity. These findings have led to research on understanding the processes in which HERV-W could be contributing to MS, with a focus on neuroinflammation and neurodegeneration. Interestingly, HERV-W env is largely expressed in macrophages and microglia and is associated with inflammatory processes and the activation of demyelinating sites [33,44]. In vitro studies have shown that HERV-W can trigger the production of inflammatory cytokines, e.g., TNF-α, IFN-β, and IL-10, through toll-like receptor 4 activation, particularly affecting monocytes and endothelial cells and leading to oligodendrocyte damage [45,46,47,48,49,50]. Moreover, HERV-W env has been shown to amplify the expression and activity of human inducible nitric oxide synthase, which contributes to demyelination, oligodendrocyte damage, and disruption of the blood–brain barrier, promoting neuronal apoptosis, axon damage, and neurodegeneration [48,51,52,53]. The antigenic properties of HERV-W in MS brain lesions stimulate microglia, potentially leading to myelinated axon damage, reflecting its implication in MS-related neurodegeneration [54]. Additionally, oxidative stress, a hallmark of neuroinflammation in MS, may perturb chromatin stability, facilitating HERV-W expression and further exacerbating disease progression [55].

Therefore, HERV-W is being considered as a therapeutic target for MS. The monoclonal antibody natalizumab, which is a common therapy for MS, has been shown to inhibit HERV-W expression in MS lymphocytes [56,57]. A monoclonal antibody and antagonist of the HERV-W env protein, Temelimab (formerly known as GNbAC1), was designed specifically for the treatment of MS [58,59,60]. A phase 2 clinical trial of Temelimab resulted in decreased levels of HERV-W expression and reduced brain atrophy and lesions [59,61,62]. Overall, there is strong evidence that HERV-W plays a critical role in the pathogenesis of MS, and current clinical trials are showing promising results in altering disease progression.

## 3. HERVs in Amyotrophic Lateral Sclerosis

Amyotrophic lateral sclerosis (ALS, also known as motor neuron disease) is a neurodegenerative disease with characteristic impairment of the motor system, which typically manifests as perturbances in gait, balance, posture, speech, and respiration [63]. Other clinical subtypes of ALS include progressive bulbar palsy, progressive muscular atrophy, and primary lateral sclerosis [63]. Epidemiologically, ALS is commonly diagnosed in adults between 55 and 75 years of age and is estimated to affect 4–8 individuals per 100,000 worldwide [64,65]. ALS presents significant health challenges, with a lack of effective treatment strategies and a poor prognosis of an average three-year survival rate post-diagnosis, primarily due to respiratory complications [66].

ALS neuropathology primarily involves aggregations of ubiquitinated cytoplasmic trans-activation response DNA-binding protein 43 (TDP-43) [67]. Although currently unknown, studies have posited that abnormal TDP-43 inclusion formation is involved in the neurodegeneration of CNS regions involved in motor control, including the primary motor cortex, upper and lower corticospinal motor neurons, the brainstem, and the anterior horn of the spinal cord [65]. Preliminary associations between dysregulated retrotransposon activity and ALS were first proposed in human serum. Andrews et al. [68] showed elevated levels of cell-free serum reverse transcriptase in patients with diagnosed ALS compared to healthy control populations [68]. Similarly, Steele et al. [69] showed elevated reverse transcriptase activity in patients with ALS compared to healthy controls. Since then, numerous studies have investigated the potential role of retrotransposons in ALS, particularly in HERV-K.

HERV-K env transcripts were found to be elevated in ALS patient serum compared to healthy controls [70]. Consistent with this, several studies have shown increases in the level of antibodies specific to HERV-K env peptides in ALS serum and CSF [71,72,73]. Further studies have verified the elevation of HERV-K in ALS sera, which has garnered interest in HERV-K as a blood biomarker for the diagnosis of ALS [70,71,72,73]. Extending from serum work, Li et al. [74] assessed HERV-K env levels in plasma-derived neuronal extracellular vesicles, but found no significant difference between ALS patients and healthy controls. However, interestingly, the levels were significantly higher in later stages of ALS compared to early stages. Therefore, HERV-K could be considered as a biomarker for ALS prognosis [74].

To date, there is only one seminal animal study investigating the pathological role of HERV-K in ALS. Li et al. [75] demonstrated the development of progressive motor dysfunction in transgenic mice overexpressing neuronal HERV-K env, mimicking ALS-like symptoms. Specifically, the mice displayed a reduced ability to travel long distances and an increased rate of fatigue and weakness in limb and spinal muscles, and developed progressive respiratory complications [75]. Histological analysis of the brains revealed neurodegeneration in the motor cortex. When put together, this study provided strong evidence to support the detrimental contribution of HERV-K to ALS [75].

Studies on post-mortem human brain tissues have shown a potential pathological role of HERV-K in ALS. Specifically, several studies have shown elevated expression of HERV-K pol, env, and gag levels in ALS brains compared to controls [75,76,77]. However, in contrast, several studies have shown no significant difference in HERV-K loci, including env, gag, and pol, in ALS brains and spinal cords compared to controls [78,79]. Therefore, further investigation in this area is necessary. Moreover, clinical studies have demonstrated a potential role of HERV-K in ALS neuropathology. Bowen et al. [80] revealed that in patients with HIV that subsequently developed ALS, the use of antiretroviral therapy reversed motor deficits or slowed down neurological symptoms. The Lighthouse clinical trial, conducted by Gold et al. [81], demonstrated that the antiretroviral drug Triumeq reduced HERV-K levels after 24 weeks of treatment. The revised amyotrophic lateral sclerosis functional rating scale (ALSFRS-R) scores were also reduced in these patients, demonstrating the beneficial effect of this antiretroviral drug on ALS [81,82].

**Figure 2:**
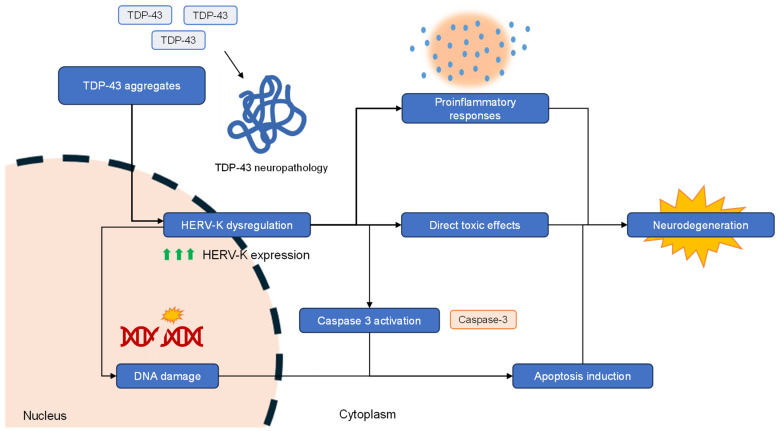
A pictorial presentation of the proposed mechanism of HERV-K mediated neurodegeneration in amyotrophic lateral sclerosis.

TDP-43, the pivotal pathogenic protein in ALS brains, has been pathologically linked to HERV-K in the study of ALS pathogenesis. TDP-43 is thought to act as a transcriptional factor for HERVs, or alternatively, impair silencing systems that usually repress retrotransposon activity, resulting in dysregulation of HERV activity [83,84,85]. Several studies have shown a potential role of TDP-43 in aberrant induction of HERV-K activity using neuronal cell models, demonstrating that TDP-43 overexpression significantly increased HERV-K env levels [70,73,75]. Interestingly, Ibba et al. [86] demonstrated that specific silencing of the HERV-K env gene interfered with TDP-43 mRNA and protein expression, providing a different insight that suggests a bidirectional relationship between HERV-K and TDP-43 pathology. Several studies have attempted to elucidate the mechanism of TDP-43 and HERV-K-dependent neurodegeneration (Figure 2). In one study, it was shown that TDP-43-dependent upregulation of gypsy, the Drosophila analogue of HERV, was related to neurodegenerative-promoting processes, including DNA damage, caspase-3 induction, and increased apoptosis [84,87], and these changes were shown to spread intercellularly [87,88]. In other studies, HERV-K was shown to cause toxicity to neurons in cell and animal models [75,87,89], and promote proinflammatory responses [90,91].

## 4. HERVs in Frontotemporal Dementia

Frontotemporal dementia (FTD) is a neurodegenerative disease characterized by symptoms of altered behavior, language, personality, and executive functions [92]. FTD is a leading cause of early onset dementia (diagnosed between 45 and 65 years of age) and is estimated to affect 10–15 individuals per 100,000 [93]. There are currently no effective treatments for FTD. FTD neuropathology is defined by its protein inclusions, the most common being TDP-43, followed by tau, and rarely fused in sarcoma protein [94]. These neuropathological protein inclusions are typically found in regions of the brain involved in the control of behavior, personality, language, and executive functions, including the frontal and/or temporal lobes [95]. FTD and ALS share similar neuropathology, i.e., TDP-43 inclusions and related genetic causes [96,97,98], and are thought to exist on opposite ends of a same disease spectrum [99]. Approximately 15% of FTD cases are estimated to develop ALS symptoms, and vice versa [100]. Very little is known about the involvement of HERV-K in FTD. To date, Phan et al. [70] is the only study to explicitly investigate the role of HERV-K in FTD. The study revealed that HERV-K levels were elevated in FTD serum compared to controls, and that they were even higher than those in ALS serum. Moreover, the HERV-K levels were also elevated in the disease-affected regions of FTD brains compared to controls [70]. Interestingly, HERV-K pol protein was shown to colocalize with TDP-43 deposits in disease-affected regions of FTD brains [70]. It was speculated that HERV-K could serve as a potential diagnostic blood biomarker for FTD [70].

## 5. HERVs in Alzheimer’s Disease

Alzheimer’s disease (AD) is a predominant form of dementia, accounting for 50–75% of all cases of dementia, and is characterized by progressive memory loss and cognitive decline [101,102]. More than 55 million people are affected with AD worldwide, with an expected increase to 78 million by 2030, representing a huge health burden globally [103]. The majority of AD cases are sporadic, with less than 5% being familial [104]. The pathological hallmarks of AD are extracellular amyloid-β plaques and intracellular neurofibrillary tangles composed of hyperphosphorylated tau [102]. Despite much research, targeted therapies against the two pathologies have been largely unsuccessful in reversing the disease or halting its progression. Alu, a SINE, has been implicated in AD pathology, with structural changes to Alu elements within the TOMM40 gene leading to translocation disruptions, mitochondrial stress, inflammation, and mitophagy, the disease processes that occur prior to macroscopic pathologies [105]. Several studies have indicated that increased DNA methylation in AD brains may play a role in the activation of TEs [106]. Tau has been shown to induce global chromatin relaxation, DNA double-stranded breaks, and abnormal transcription of heterochromatic genes, which may activate TEs and induce neurodegeneration [107,108]. Analysis of tau transgenic Drosophila models and AD transcriptomes showed that tau pathology induces retrotransposon activity via heterochromatin relaxation [109]. Neurofibrillary tangle burden is associated with elevated HERV-Fc1 transcripts, suggesting tau pathology could activate HERV transcription [109]. Interestingly, in a tauopathy mouse model, TE derived proteins and TE DNA copy number were increased in the brain compared to control mice, suggesting a pathological relationship between tauopathy and TE transposition [110]. RNA sequencing analysis of AD and progressive supranuclear palsy, another tauopathy, cortices showed elevated levels of HERV-H, HERV-K, and HERV-L compared to healthy controls, further suggesting that HERV dysregulation is related to tauopathy [111].

Analysis of AD brain transcriptomes showed that HERV-K expression was elevated compared to controls, and that HERV-K was the most expressed and differentially upregulated TE in AD [112]. Moreover, reduced cognition in the year prior to death has been associated with increases in certain HERVs in AD brains [109]. More recently, Dawson et al. [113] used Telescope, a tool enabling accurate estimation of HERV expression at specific loci, to identify 698 HERVs that were differentially expressed in AD brains compared to control brains. Those that were over-represented included HML4, HARLEQUIN, HERV-Fc1, HERV-K11D, and HERV-K11, and those differentially expressed included HERV-K, HERV-W, HML3, HML2, HML4, and HML6 [113]. Since HMLs belong to the broader HERV-K group, HERV-K is greatly over-represented in AD brains. Furthermore, HERV-K transcripts in CSF were significantly elevated in AD compared to controls [112].

Analysis of the AD temporal cortex transcriptome showed a correlation between increased HERV-K and toll-like receptor 8 (TLR8) RNA expression, suggesting a pathological link between HERV-K and neuroinflammation [112]. However, this relationship was shown to be absent at the HERV-K family level, and only a subset of HERV-K loci was significantly correlated to TLR8 and the interferon genes involved in innate immune activation [113]. Furthermore, gene enrichment analysis showed that HERVs are most proximal to genes involved in cell adhesion and immune response pathways [113]. Interestingly, in AD brain tissues, HERV-W levels were only detected when tumor necrosis factor-α was also present, further implicating the potential relationship between HERVs and inflammation [114].

Licastro and Porcellini [115] hypothesized that microbial infection of the CNS has the potential to initiate inflammation and neurodegeneration, as well as stimulate HERVs in the brain. They suggested that this activation may then act as continuous abnormal stimulation of inflammatory processes, ultimately contributing to the neurodegeneration observed in AD [115]. Additionally, Evering et al. [116] proposed a pathological cycle in which neuronal protein aggregation in AD leads to dysregulation of TE expression in affected neurons, which results in TE gene products and reverse transcription, triggering innate immune signaling. They suggested that this leads to microglial activation, which releases inflammatory cytokines and induces reactive astrocytes, leading to the release of neurotoxins. Despite these proposed hypotheses, many questions remain regarding the mechanisms by which HERVs induce inflammation and neurodegeneration, or whether inflammation stimulates HERV activation. As it currently stands, the role of HERVs in AD pathogenesis is unclear, and much more research is needed.

## 6. HERVs in Parkinson’s Disease

Parkinson’s disease (PD), the second most common neurodegenerative disease, had a worldwide prevalence of 8.5 million in 2017, representing 1–2% of individuals over 65 [117,118]. It is the fastest growing neurological disorder, with the prevalence expecting to double over the next 20 years, representing a rising burden to society [119]. The majority of cases are sporadic, with approximately 5–10% of patients having familial PD [120]. PD is characterized by the loss of dopaminergic neurons, classically in the ventrolateral tier of the substantia nigra pars compacta (SNpc) [121]. Another hallmark of PD is the presence of α-synuclein aggregates, known as Lewy bodies, in neurons [120]. α-Synuclein aggregates are also found in glial cells, including astrocytes, microglia, and oligodendrocytes [122]. The loss of dopamine in PD leads to severe motor symptoms of rest tremor, rigidity, and bradykinesia [123]. Non-motor symptoms include cognitive impairment, dysautonomia, and neurobehavioral disorders [123]. There are no disease-modifying treatments that reduce PD progression, and drugs targeting the dopamine deficit only alleviate motor symptoms. A deeper understanding of PD pathogenesis is essential to establish targets for disease modification [122].

Despite PD being a high burden disease, there have been only a few studies investigating HERVs in PD. Gordevičius et al. [124] found that HERVs are upregulated in the pre-frontal cortex, pre-frontal neurons, and the blood in late stages of PD. They also showed that genes that correlate with HERV expression are enriched in the apoptosis and cellular respiration pathways. In a PD mouse model with increased synucleinopathy, ERVs were shown to be upregulated, suggesting a link between α-synuclein pathology and ERV activation. Whole-genome sequencing indicated that polymorphic HERV-K could contribute to neurologic and immunologic phenotypes observed in PD [125]. Finally, the only study to analyze HERV expression in PD showed no detection of HERV-K pol transcripts in PD brain tissues [76], suggesting the alterations in genomic HERVs may not translate into proteins.

In mouse models of PD, LINE-1 expression was observed in SNpc dopaminergic neurons and linked to oxidative stress, DNA damage, and neurodegeneration of the neurons [126]. More recently, whole-genome sequencing identified SINE-VNTR-Alu retrotransposons, which were shown to be associated with PD progression [127]. Additionally, elevated retrotransposition events of LINE-1 were detected in PD blood, revealing a potential association of LINE-1 with PD risk and disease progression [127]. Furthermore, transcriptional analysis of PD pre-frontal cortex suggested that the activation and dysregulation of TEs are a dynamic and common characteristic of PD [124]. More specifically, upregulation of TEs was evident in the brain regions associated with disease symptom onset, and this upregulation was less evident in prodromal patients [124]. The activation of TE in blood was shown to be highest at the time of diagnosis, which could be used to develop biomarkers for PD diagnosis and prognosis [124].

A recent study using a human microglial cell model showed that viral particles from the SARS-CoV-2 virus mediated microglial inflammasome activation, and this was enhanced in the presence of α-synuclein fibrils, providing evidence that viral particles are capable of causing PD-like neuroinflammation [128]. Furthermore, studies suggest that HERV-K env could be involved in inducing reactive astrogliosis, the abnormal proliferation of astrocytes, and the activation of other glial cells [75,129]. These studies are significant, as they demonstrate the potential involvement of HERV-K in neuroinflammation similar to that observed in PD. However, the involvement of HERV-K in the pathophysiology of PD remains unresolved, warranting further investigation.

## 7. Conclusions and Perspective

This review presents a comprehensive summary of HERV involvement in neurodegenerative diseases. From current evidence, stemming from preliminary serological work, preclinical animal and human studies, and clinical studies/trials, research has revealed the interesting potential of HERV involvement in neurodegeneration. Classically, the common prominent neurodegenerative diseases with more established associations to aberrant HERV activity are MS and ALS. Understanding of HERV-W has garnered convincing evidence marking it as a potential driver of MS, with current clinical trials targeting HERV-W. On the other hand, HERV-K is the leading retrotransposon associated with ALS. Despite overall data supporting HERV-K as a potential diagnostic biomarker and therapeutic target for ALS, its exact role in the development and progression of the disease is unclear. Extending from this, interest in the field has grown regarding the role of HERVs in other neurodegenerative diseases, including FTD, AD, and PD. In FTD, a TDP-43 proteinopathy with similar neuropathology to ALS, HERV-K has been shown to be a potential driver of neurodegeneration. The role of HERV-K, HERV-Fc1, and HERV-W in AD pathogenesis has been explored, with interesting leads that require further studies. Research on HERV involvement in PD is only preliminary at this stage, with no specific HERV investigated in PD development or progression. Overall, the research thus far has gathered significant and promising data supporting the involvement of HERVs in neurodegeneration, with positive results from clinical trials, such as Temelimab in MS. These have provided the groundwork for further research in developing novel therapeutics and biomarkers for neurodegenerative diseases.

However, despite these promising results, research into individual HERVs in neurodegenerative diseases has yielded some inconsistent and conflicting results, likely due to methodological differences and variations in sampling cohorts, reflecting the need for HERV-specific validated assays that can be easily replicated in multiple settings [130]. Although some HERV-related disease mechanisms have been proposed, it is unclear whether the upregulation of different HERVs is a cause or an effect of disease. Furthermore, common weaknesses of research in this field include small sample sizes and heterogeneity of brain tissues used, which render low statistical significance. Future studies need to also focus on examining longitudinal cohorts to provide more reliable information on patterns of HERV expression related to disease progression. Additionally, comparisons of clinical phenotypes and neuropathology with HERV levels would be beneficial in understanding whether HERV is differentially affected in certain disease states. Correlating HERVs in biofluids to established biomarkers, such as total tau in AD plasma [131], would be valuable for biomarker panel development. Furthermore, understanding both normal and abnormal function of HERVs in cell and animal models would allow greater understanding of the disease mechanisms at play and the downstream consequences of HERV expression. Current improvements in sequencing technologies, with the ability to detect longer reads and repetitive genomic regions, will simplify the detection of HERV transcripts and novel genomic insertions [3]. In addition, the recent development of single-cell RNA sequencing provides the potential to differentiate HERV expression in different cell types, which may lead to therapeutic strategies that target disease prior to pathology onset [116].

HERVs constitute approximately 8% of the human genome. They are present in all human cells as a result of the integration of exogenous retroviruses into mammalian germ cells millions of years ago. Most HERVs are silent or inactive because of genomic rearrangements or epigenetic changes. Once described as junk DNA because of their perceived lack of function or purpose, they have now been shown to be associated with critical roles in the host, including embryogenesis, cancer, and autoimmune and neurological diseases. The importance of HERVs is underscored by the fact that they provide approximately 320,000 binding sites for transcription factors in the human genome [132]. They can also contribute to a number of activities that affect gene regulation and the formation of chimeric transcripts, resulting in genomic instability. Understanding the full implication of HERVs has been difficult because of the limitations of diagnostic tools, and the fact that approximately 40–90% of the metagenomic viral reads do not align to any reference viral sequences [133].

In summary, while some progress has been made in the understanding of HERVs in neurodegenerative diseases, much research is needed to determine the underlying pathomechanisms linking HERVs to proteinopathies and to develop HERV therapies and biomarkers for neurodegenerative diseases.

## Data Availability

Not applicable.
